# A brief grief over bowel relief

**DOI:** 10.12688/f1000research.2-26.v1

**Published:** 2013-01-29

**Authors:** Kamalpreet S Parmar, Malvinder S Parmar

**Affiliations:** 1 University of Queensland, Brisbane, QLD 4072, Australia; 2 Northern Ontario School of Medicine, Laurentian & Lakeland Universities, Ontario, P3E 2C6, Canada

## Abstract

Oral sodium phosphate (OSP) solution is commonly used as bowel purgative before colonoscopy. Its safety has recently been questioned with several reports of acute renal failure and chronic kidney disease following its use. All of the cases reported are following bowel preparation for colonoscopy. I present a case of acute renal failure following OSP solution given to relieve constipation. This report further highlights the dangers of OSP and the importance of caution and careful monitoring when OSP solution is used as a cathartic, or for bowel preparation before colonoscopy.

## Case report

A 72-year-old woman with essentially unremarkable past medical history fell and sustained back injury and was noted to have a T _11_ compression fracture without any neurovascular compromise. The patient received Tylenol#3 for pain relief and was sent home. A few days later, she returned with ongoing vague lower back and abdominal discomfort and was noted to be constipated. Tylenol#3 was stopped and she was given a laxative - oral sodium phosphate solution (OSP, 45 ml, *Pharmascience, Montreal, Canada*) to treat constipation.

Three days later, she returned to the local emergency department with feeling of generalized weakness, numbness around her lips, ongoing vague abdominal discomfort and nausea, but denied vomiting or diarrhea. Her intake had been poor since the fall and she noted decreased urine output. There is no history of diabetes or hypertension. Her medication was rabeprazole 20 mg a day and acetaminophen as required.

Investigations at the local emergency department revealed low hemoglobin of 109 g/L, normal white blood cell count WBC of 4.5, elevated blood urea nitrogen BUN of 9.4 mmol/L with serum creatinine of 345 μmol/L, and serum potassium of 3.4 mmol/L. The old records showed that her BUN was 6.1 mmol/L with serum creatinine of 74 μmol/L in December 2007. She was transferred for further management of acute renal failure.

Physical examination was remarkable for a woman of stated age with mild decreased skin turgor, blood pressure of 106/60 mmHg without orthostatic changes and regular rate of 72 beats per minute. Lungs were clear and heart sounds were normal. Abdominal examination revealed a soft abdomen with mild diffuse tenderness without rebound. There were no masses, renal angle fullness or tenderness. There was mild tenderness in the lower thoracic area. There was no pedal edema and neurological examination was non-focal. She had a Foley catheter with small amount of concentrated urine in the bag.

Investigations in our emergency department revealed low hemoglobin of 112 g/L, normal WBC of 4.9, elevated BUN of 9.4 mmol/L with serum creatinine of 419 μmol/L, serum potassium of 3.4 mmol/L, low serum calcium of 1.85 mmol/L (2.02–2.60 mmol/L) with serum albumin of 36 g/L, low ionized calcium of 0.85 mmol/L (1.15–1.29 mmol/L) and elevated phosphate of 3.68 mmol/L (0.87–1.45 mmol/L) and creatine kinase [CK] of 349. A urinalysis showed a concentrated urine with specific gravity of >1.030, 1+ protein and trace of blood with few white and red blood cells with few hyaline casts. Random urine sodium was 64 mmol/L with urine creatinine of 5330 μmol/L. A urine culture was negative. An abdominal ultrasound showed normal size kidneys without obstruction. The hospital course is shown in Table 1.

**Table 1.** Showing the hospital course of the patient.

	Normal range	9-months before	Day 1	Day 2	Day 5		Day 10	Day 21
BUN	2.6–7.7 mmol/L	6.1	9.9	11.3		**Hemodialysis × 2**		6.4
Serum creatinine	35–97 μmol/L	74	419	486	675		200	98
Serum potassium	3.6–5.0 mmol/L		3.6					3.8
Serum calcium	2.02–2.60 mmol/L		1.85	1.66	1.92		2.20	2.24
Ionized calcium	1.15–1.29 mmol/L		0.85	0.94			1.07	1.16
Serum albumin	38–46 g/L		33					38
Serum Phosphate	0.87–1.45 mmol/L		3.68	3.59	3.39		1.12	1.18

**Figure 1.**
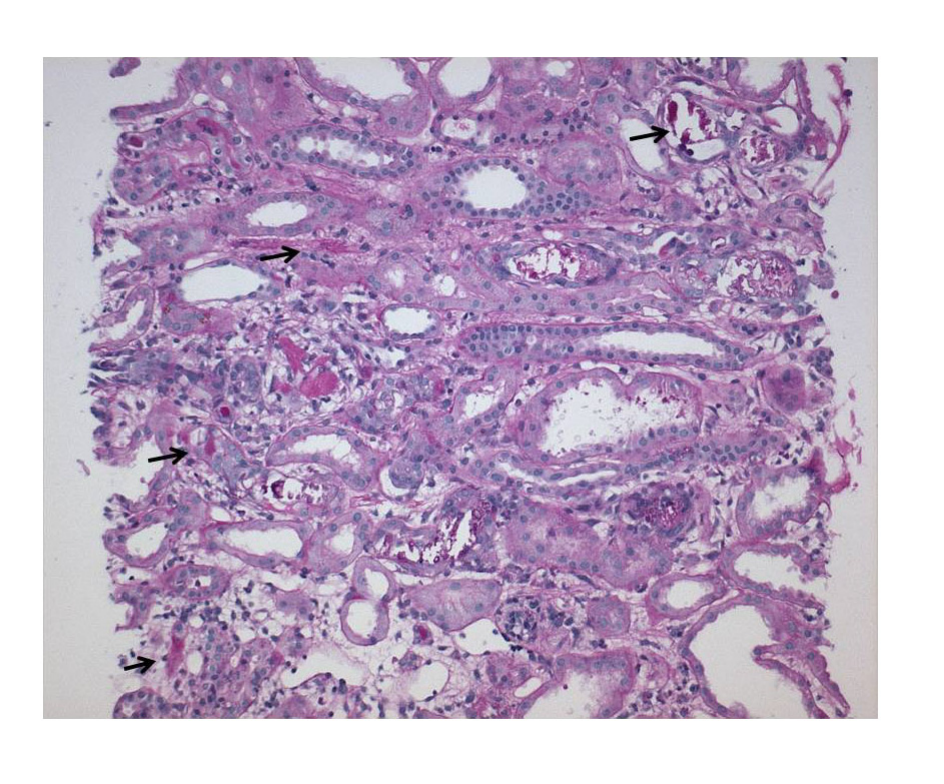
Kidney biopsy – showing numerous tubules with calcification (black arrows) (PAS stain, magnification x100) [Courtesy: Andrew Herzenberg (deceased) and John Rohan, University Health Network, Toronto, Ontario, Canada].

This patient presented with acute kidney injury (AKI) and the differential diagnosis included ischemic acute tubular necrosis (poor intake, decreased skin turgor, FE _Na_ of 3.78%), rhabdomyolysis (history of fall, elevated phosphate). The likelihood of vasculitic process was low in view of bland urine sediment and negative antinuclear and anti-neutrophilic cytoplasmic antibodies. However, significant hyperphosphatemia and hypocalcemia within 72-hours of standard dose (45 ml) of OSP [ *21.6 gm of monobasic sodium phosphate monohydrate and 8.1 gm of dibasic sodium phosphate heptahydrate*] suggests the high probability of acute phosphate nephropathy (APN) ^1^ that results from deposition of calcium-phosphate crystals in renal tubules and parenchyma (nephrocalcinosis) ^2^. A kidney biopsy confirmed findings of acute phosphate nephropathy with acute tubular necrosis ( Figure 1). She required supportive dialysis treatment twice.

## Discussion

OSP solution is commonly used as a bowel purgative before colonoscopy ^3^. Its safety has recently been questioned ^1– 5^ with several reports of AKI and chronic kidney disease following its use. All of the cases reported occurred after bowel preparation with OSP for colonoscopy but AKI in this woman occurred after its use to relieve constipation. Decreased kidney function, use of renin angiotension aldosterone system (RAAS) blockers and older age and female gender are the most probable risk factors for APN, but other contributing factors are - use of non-steroidal anti-inflammatory agents, diuretics, history of hypertension, diabetes or heart failure ^3– 5^. Women, because of their smaller body mass, are more sensitive to fluid loss. Adequate fluid intake ^4^ is important to prevent AKI when OSP is used as a bowel purgative. Poor oral intake since the fall in this woman likely contributed to both ATN and APN after OSP use. Although most patients recover renal function, some may have persistent chronic kidney disease ^4^.

This report further highlights the need for vigilance when using OSP solutions for bowel preparation or to relieve constipation. Alternative solutions should be considered, especially in elderly and high-risk individuals ^5^.

**Key Points**

1. Consider acute phosphate nephropathy in the presence of acute renal dysfunction, hyperphosphatemia and hypocalcemia.

2. Consider alternate agents to oral phosphate solutions for bowel preparation or for relief of constipation, especially in elderly patients and in patients with chronic kidney disease.

3. Ensure adequate hydration, if and when these agents (OSP) are used.
