# Supplementary material for: A brief grief over bowel relief
Source: F1000Res. 2013 Jan 29;2:26. [Version 1] doi: 10.12688/f1000research.2-26.v1 (PMC3894798; doi:10.12688/f1000research.2-26.v1)
Supplement: Peer review history

# Peer review: Referee response for version 1

**Published:** 2013-11-05

**Authors:** Luan D. Trong

**Affiliations:** 1 Houston Methodist Research Institute, Houston, TX, USA

In this manuscript entitled “A Brief Grief over Bowel Relief,” by K.S. Parmar and M.S. Parmar, the authors describe a patient with acute renal failure after oral sodium phosphate administration for constipation.

As the authors mentioned, this type of renal failure is almost always secondary to administration of sodium phosphate for colonoscopy. This report expands the possible complications of oral sodium phosphate in a new clinical context, i.e. treatment for constipation. It is an important finding both clinically and pathologically.

A few minor suggestions:

1. The quality of the renal biopsy illustration can be improved. The picture should be sharper with better contrast and the areas of tubular calcification should be better illustrated.
2. In the Discussion, the dose as well as the drug schedule of oral sodium phosphate for colonoscopy should be reviewed and compared with those in this case. This should highlight better the clinical context in which oral sodium phosphate as a treatment for constipation can cause acute renal failure.

I have read this submission. I believe that I have an appropriate level of expertise to confirm that it is of an acceptable scientific standard.

# Peer review: Referee response for version 1

**Published:** 2013-03-18

**Authors:** Christina Yuan

**Affiliations:** 1 Walter Reed Army Medical Center, Washington, DC, USA

This is a case report of acute kidney injury (requiring dialysis) after use of OSP for constipation in an elderly woman. The patient had a kidney biopsy demonstrating findings consistent with acute phosphate nephropathy. This is a valuable reminder that AKI secondary to OSP may occur independently of the indication for OSP prescription.

I have read this submission. I believe that I have an appropriate level of expertise to confirm that it is of an acceptable scientific standard.
